# Nature, availability, and utilization of women-focused cardiac rehabilitation: a systematic review

**DOI:** 10.1186/s12872-021-02267-0

**Published:** 2021-09-23

**Authors:** Taslima Mamataz, Gabriela L. M. Ghisi, Maureen Pakosh, Sherry L. Grace

**Affiliations:** 1 Faculty of Health, York University, Bethune 368, 4700 Keele Street, Toronto, ON M3J 1P3 Canada; 2 KITE-Toronto Rehabilitation Institute, University Health Network, University of Toronto, Toronto, ON Canada; 3 Library & Information Services, Toronto Rehabilitation Institute, University Health Network, University of Toronto, Toronto, ON Canada; 4 Peter Munk Cardiac Centre, KITE-Toronto Rehabilitation Institute, University Health Network, University of Toronto, Toronto, ON Canada

**Keywords:** Cardiac rehabilitation, Women, Systematic review, Healthcare utilization, Adherence, Heart diseases, Secondary prevention, Access, Satisfaction, Health services delivery

## Abstract

**Background:**

Women do not participate in cardiac rehabilitation (CR) to the same degree as men; women-focused CR may address this. This systematic review investigated the: (1) nature, (2) availability, as well as (3a) utilization of, and (b) satisfaction with women-focused CR.

**Methods:**

Medline, Pubmed, Embase, PsycINFO, CINAHL, Web of Science, Scopus and Emcare were searched for articles from inception to May 2020. Primary studies of any design were included. Adult females with any cardiac diseases, participating in women-focused CR (i.e., program or sessions included ≥ 50% females, or was 1-1 and tailored to women’s needs) were considered. Two authors rated citations for inclusion. One extracted data, including study quality rated as per the Mixed-Methods Assessment Tool (MMAT), which was checked independently by a second author. Results were analyzed in accordance with the Synthesis Without Meta-analysis (SWiM) reporting guideline.

**Results:**

3498 unique citations were identified, with 28 studies (53 papers) included (3697 women; ≥ 10 countries). Globally, women-focused CR is offered by 40.9% of countries that have CR, with 32.1% of programs in those countries offering it. Thirteen (46.4%) studies offered women-focused sessions (vs. full program), 17 (60.7%) were women-only, and 11 (39.3%) had gender-tailoring. Five (17.9%) programs offered alternate forms of exercise, and 17 (60.7%) focused on psychosocial aspects. With regard to utilization, women-focused CR cannot be offered as frequently, so could be less accessible. Adherence may be greater with gender-tailored CR, and completion effects are not known. Satisfaction was assessed in 1 trial, and results were equivocal.

**Conclusions:**

Women-focused CR involves tailoring of content, mode and/or sex composition. Availability is limited. Effects on utilization require further study.

**Supplementary Information:**

The online version contains supplementary material available at 10.1186/s12872-021-02267-0.

## Introduction

Cardiovascular diseases (CVDs) are among the leading causes of morbidity and mortality in women globally [1]. It has been well-recognized that more attention needs to be paid to women’s cardiovascular health due to their poorer outcomes than men, such as more disability, hospitalization and early mortality [2]. This is likely due to the ways CVD risk factors are manifested differently in women (e.g., tobacco use and oral contraceptives, diabetes more hazardous, low socioeconomic status more preponderant as well as depression [3, 4]). Moreover, diagnostic tests are less sensitive in women [5], which is related to the fact that they often have different forms of CVD (e.g., heart failure with preserved ejection fraction, myocardial infarction with non-obstructive coronary arteries, coronary artery dissection and vasospasm) [6], and results in women being diagnosed at later stages of disease than men [5]. Finally, women have a greater burden of some comorbidities than men [6].

Cardiac rehabilitation (CR) is a standardized model of secondary preventive care proven to mitigate this burden. It offers the core components of risk factor management, structured exercise, patient education and psychosocial counselling [7], in a clinical or home-based setting, offered on average over 4 months [8]. It is established that CR participation results in approximately 20% lower mortality and morbidity [9], improved quality of life [10], and is cost-effective [11]. Indeed, CR is recommended for women in clinical practice guidelines [12].

Despite these benefits, CR is under-utilized globally [13], especially in women. Meta-analyses show fewer women than men are referred to CR programs (39% women vs. 49% men) [14]. Even after being referred, women are less likely than men to enroll (38.5% vs. 45.0%) [15] and adhere (64.2% vs. 68.6%) [16] to prescribed sessions. Reasons for women’s under-utilization are multifactorial, including lack of awareness due to less provider endorsement [17], transportation barriers, family obligations and experiencing exercise as tiring or painful, often related to their greater comorbidities [18, 19]. Women also have different CR preferences than men, and many of their preferences are not well-met [20–22]. For example, women prefer engaging in yoga or dance as forms of exercise rather than using treadmills or cycle ergometers, and they prefer not being rushed, crowded or weighed (the latter two related to desire for privacy).

To address these gender-specific issues, “women-focused” (also termed “gender-tailored” [which we consider to mean that content is adapted to women’s needs and preferences], or “women-only” [meaning the sex composition of CR programming only comprises women] in the literature, among other terms) sessions or full programs have been developed, to attempt to address their barriers and meet their unique needs [23, 24]. Indeed, it is important that in the field we start to better delineate what kind of women-focused CR is being delivered, in terms of how or whether the following characteristics/elements are present (not mutually-exclusive): (a) women-only or mostly women (also considering individually-delivered care [in-person or remote] which is inherently “women-only”, particularly if the healthcare provider(s) is female), (b) content tailored to women (and specifically what and how), and/or (c) some versus the full program is either (a) and/or (b) (e.g., peer support sessions for women, a separate education session for women; Table 1).

**Table 1:** Characteristics of women-focused CR (N = 28)

Study First Author’s Last Name (citations), Year first publication, Country; quality^‡^	Women-Focused CR Intervention Features			
	Dose (# of sessions [freq/wk x # wks]; delivery (multidisciplinary team—y/n); open access materials; group size; phase	Exercise (mode, supervised vs not [or both], intensity, session duration [min]); RT (y/n)	CR components other than exercise (pt education, risk factor management [tobacco cessation, blood pressure, lipids], psychosocial, nutrition counselling, other); mode of delivery (e.g., f2f, tech)	Gender-tailoring (n or y; if y, specify); sessions or whole program tailored; theoretical basis; proportion of women in sessions (100% if all unsupervised)
Andersson et al. [42] 2010, Sweden; quality: 4/5	Dose: 33 sessions (10 days residential followed by 5 inpatient days after 2 months, then twice yearly for 2 inpatient days from 2^nd^ year to 5^th^ year); delivery: cardiologist, psychologist, psychiatrist, dietitian, physiotherapist (multidisciplinary team: y); open access materials: no; group size: 6–10; phase II	Mode: walking with or without stick, aerobics, yoga, QiGong and water-aerobics; supervised: y[both]; intensity: NR; session duration: NR; RT: no	other components: tobacco cessation, dietary counseling, relaxation/stress management; mode of delivery: f2f: y; tech: cassette tapes	Gender-tailoring: no; theoretical basis: no; proportion of women in sessions: ≥ 50%
Arthur et al. [43] 2007, Canada; quality: 4/5	Dose: 49 sessions (after initial assessment, twice weekly for 8 weeks with only aerobic exercise, then twice weekly for 16 weeks combined aerobic and strength training); delivery: certified kinesiologist, physician (multidisciplinary team: y); open access materials: no; group size: NR; phase II	Mode: walking on treadmills, stationary cycles, arm ergometers, stair climbers; supervised: y[both]; intensity: gradually increasing from 40 to 70% of functional capacity based on GXT results; session duration: average 60 min; RT: y (2 times of 8–10 repetitions starting at 30% increasing gradually to 70% of 1 RM for upper body and 2 times of 10–12 repetitions with 50–70% of 1 RM for lower body)	other components: comprehensive CR with tobacco cessation, nursing education and support, dietary counselling; mode of delivery: f2f: y; tech: no	Gender-tailoring: y (not specified); theoretical basis: no; proportion of women in sessions: ≥ 50%
Asbury et al. [44] 2008, UK; quality: 4/5	Dose: 16 sessions (standard 8 week CR comprised of 1×/wk outpatient exercise and 1×/wk home-based sessions); delivery: registered nurse, physician, cardiologist (multidisciplinary team: y); open access materials: no; group size: NR; phase: III	Mode: NR; supervised: y[both] intensity: gradually increasing from 60 to 75% of age-predicted HR reserve; session duration: 80 min; RT:NR	other components: varied and not specified; mode of delivery: f2f: y, tech: y (phone calls)	Gender-tailoring: no; theoretical basis: no; proportion of women in sessions: 100%
Azad et al. [45] 2012, Canada; quality: 3/5	Dose: 12 sessions (twice per wk for 6 wks); delivery: physician, nurse, physiotherapist, occupational therapist, dietician, pharmacist, and social worker. (multidisciplinary team: y); open access materials: no; group size: NR; phase II	Mode: NR; supervised: y[both]; intensity: started with lowest intensity/duration then gradually increased with last exercise interval as the highest intensity; exercise prescription based on RPE scale and THR by 2–5 min assessment walk; session duration: 30 min; RT: no	other components: education, counseling, and dietary management; mode of delivery: f2f: y; tech: y (phone call at 30th wk)	Gender-tailoring: no; theoretical basis: no; proportion of women in sessions: ≥ 50%
Beckie et al. [46–53] 2010, USA; quality: 5/5	Dose: 36 sessions (3 times/wk × 12 wks); delivery: female nurses, exercise physiologist, clinical psychologist, clinical nurse specialist (multidisciplinary team: y); open access materials: no; group size: NR; phase: II	Mode: treadmill, walking, cycling, or rowing; supervised: y[both]; intensity: 60–85% of maximal HR with gradual increase in intensity; session duration: 60 min; RT: y (wall-pulleys and hand weights)	other components: two 1 h individualized MI counseling and 10 psychoeducational classes focusing on CHD risk factor modification, social support, relaxation exercises, and one 30 min dietitian consultation; mode of delivery: f2f: y; tech: no	Gender-tailoring: no; theoretical basis: y (transtheoretical model and MI for behavior change); proportion of women in sessions: 100%
Chou et al. [54] 2016, Canada; quality: 4/5	Dose: 24 sessions (1×/wk for 24 wks); delivery: cardiologist, registered nurse, kinesiologist, fitness instructor, social worker, psychiatrist, dietitian; (multidisciplinary team: y); open access materials: no; group size: NR; phase: II	Mode: using aerobic machines in the centre; supervised: y; intensity: THR 50–70% of the HR reserve based on entrance exercise test; session duration: 60 min; RT: y (light weight 2–12 lbs, and advised not to lift greater than 20 lbs)	other components: education sessions (20 min per wk) contained heart healthy nutrition, risk factors, treatment of heart disease and stress management, psychosocial counselling, peer group support; mode of delivery: f2f: y; tech: no	Gender-tailoring: y (SCAD-CR program was developed for women after a SCAD event emphasizing management of women’s heart disease); theoretical basis: no; proportion of women in sessions: 100%
Clark et al. (Women Take Pride trial) [55–58] 2003, USA; quality: 5/5	Dose: 6 sessions (initial orientation, then 1×/wk × 5 wks); delivery: nurse health educator, peer leader (multidisciplinary team: y); open access materials: https://cmcd.sph.umich.edu/research-program-areas/women-take-pride/; group size: 6–8; phase: II	Mode: NR; supervised: hybrid (single orientation session then at home); intensity: NR; session duration: 120–150 min; RT: NR	other components: self-education on risk factor management, dietary advice and self-management of stress; mode of delivery: f2f: y, tech: y (phone calls)	Gender-tailoring: y (A 4-week education and behavior modification program designed to improve heart disease management by enhancing women’s self-regulation. The program was called “Women take PRIDE” because it focused on **P**roblem selection, **R**esearching one’s daily routine, Identifying a self-management goal, Developing a plan for goal attainment, and Establishing a reward.); theoretical basis: y (social cognitive theory; self-regulation); proportion of women in sessions: 100%
Davidson et al. [59] (HAWP-Heart Awareness for Women Program) 2008, Australia; quality: 5/5	Dose: 6 sessions (once per wk for 6 wks); delivery: CR nurse, nurse researcher, health professional-facilitator. (multidisciplinary team—y); open access materials: no; group size: 5–10; phase II	Mode: NR; supervised: y; intensity: NR; session duration: 120 min; RT: no	other components: pt education, psychosocial counselling; mode of delivery: f2f: y; tech: no	Gender-tailoring: y (The program aimed to educate women on the importance of heart health education and awareness for its prevention which empower women to manage their own heart health.); theoretical basis: y (mutual aid model); proportion of women in sessions: 100%
Eyada et al. [60] 2007, Saudi Arabia; quality: 3/5	Dose: NR; delivery: cardiologist, physiotherapist (multidisciplinary team: y); open access materials: no; group size: NR; phase: I, II, III	Mode: NR; supervised: y; intensity: NR; session duration: NR; RT:NR	other components: pt education, psychosocial; mode of delivery: f2f: y, tech: no	Gender-tailoring: no; theoretical basis: no; proportion of women in sessions: ≥ 50%
Feizi et al. [35] 2012, Iran; quality: 3/5	Dose: 26 sessions (2 instructional sessions for 60–90 min then exercise at home 3 times/wk for 8 wks); delivery: nurse researcher, physician, psychologist (multidisciplinary team: y); open access materials: no; group size: NR; phase: III	Mode: walking; supervised: hybrid [2 f2f, then rest are home-based]; intensity: 60–65% of maximal HR; session duration: 25–40 min; RT: NR	other components: pt education, psychosocial; mode of delivery: f2f: y, tech: y (wkly phone calls, Cds to practice exercise at home)	Gender-tailoring: no; theoretical basis: no; proportion of women in sessions: 100%
Gary et al. [61–63] 2003, USA; quality: 4/5	Dose: 36 sessions (3×/wk for 12 wks); delivery: nurse researcher only (multidisciplinary team: no); open access materials: no; group size: 1-1; phase: II	Mode: walking; supervised: y (individual home-based); intensity: low to moderate-intensity (at 40% intensity at the beginning then gradually increase in duration and intensity up to 60%); session duration: maximum 30 min; RT: NR	other components: pt education; mode of delivery: f2f: y, tech: no	Gender-tailoring: y (education); theoretical basis: no; proportion of women in sessions: 100%
Grace et al. (CR4HER trial) [21, 64–67] 2014, Canada; quality: 4/5	Dose: ~ 48 sessions (varied by program); delivery: physician, dietitian, kinesiologist, nurse (multidisciplinary team—y); open access materials: education materials; group size: varied; phase II	Mode: treadmill walking; supervised: y; intensity: moderate based on stress test; session duration: 60 min; RT: y	other components: pt education, stress management, risk factor management, nutrition counseling; mode of delivery: f2f: y; tech: no	Gender-tailoring: no; theoretical basis: no; proportion of women in sessions: 100%
Gunn et al. [68] 2007, Canada; quality: 3/5	Dose: 10–12 sessions (once per wk for 10–12 wks); delivery: kinesiologists, nurses, physicians (multidisciplinary team—y); open access materials: no; group size: NR; phase II	Mode: NR; supervised: y[both]; intensity: NR; session duration: 120 min; RT: y	other components: pt education, nutrition counselling; mode of delivery: f2f: y; tech: no	Gender-tailoring: y (education); theoretical basis: no; proportion of women in sessions: 100%
Heald et al. [40, 41] 2020, Canada; quality: 4/5	Dose: 25 sessions (1×/wk for 24 wks and 1 initial assessment); delivery: exercise physiologist, physician, dietitian, social worker and psychologist; (multidisciplinary team: y); open access materials: https://www.healtheuniversity.ca/en/cardiaccollege/; group size: NR; phase: II	Mode: treadmill walking, cycle ergometer; supervised: y; intensity (from 60–80% of HR reserve); session duration: 60 min; RT: y (initial weight load of 60% of 1-repetition maximum was used and then gradually increased)	other components: pt education, risk factor management, stress management, and nutrition counseling; mode of delivery: f2f: y, tech: no	Gender-tailoring: no; theoretical basis: no; proportion of women in sessions: 100%
Kennedy et al. [69] 2003, Canada; quality: 4/5	Dose: 42–56 sessions (supervised 2–3 days per wk for 7 wks, and then 4–5 days/wk unsupervised for another 7 wks); Delivery: physical therapist, dietitian, social worker (multidisciplinary team—y); open access materials: no; group size: NR; phase II	Mode: treadmill walking, cycle ergometer; supervised: hybrid (7 wks supervised then at home); intensity: 70–85% of maximal HR; session duration: 40 min; RT: y (resistance exercises on weight-training machines or using free weights)	other components: 5 education sessions addressing heart-health lifestyle topics; mode of delivery: f2f: y (and remote); tech: no	Gender-tailoring: no; theoretical basis: no; proportion of women in sessions: ≥ 50%
Madison et al. [39] 2010, UK, quality: 5/5	Dose: four modules over 4 wks; delivery: nurse researcher (multidisciplinary team: no); open access materials: no; group size: no; phase: NR (some participants attended phase III CR)	Mode: not explicitly reported but recommended to perform aerobic exercises (walking, swimming, rowing, stair climbing) 3×/wk for at least 30 min; unsupervised; intensity: NR; session duration: 30 min; RT: recommended	Other components: pt education regarding risk factors management, tobacco cessation, nutrition, PA, psychosocial and mental health activities designed to enhance self-awareness; mode of delivery: f2g: y, tech: no	Gender-tailoring: y (self-management learning modules specific for rural women with CHD); theoretical basis: y (social cognitive theory); proportion of women in sessions: 100%
Mahmoodian et al. [36] 2012, Iran; quality: 1/5	Dose: 24 sessions (3×/wk for 8 wks); delivery: NR; (multidisciplinary team: NR); open access materials: no; group size: NR; phase: II	Mode: NR; supervised: y; intensity: NR; session duration: NR; RT:NR	other components: NR; mode of delivery: f2f: y; tech: NR;	Gender-tailoring: no; theoretical basis: no; proportion of women in sessions: 100%
Price et al. 2005 [23, 70–72] Canada; quality: 5/5	Dose: 24 sessions (1×/wk for 24 wks); delivery: nurse-practitioner, cardiologist, physiotherapist, exercise specialist, respiratory therapist, registered dietitian, social worker (multidisciplinary team: y); open access materials: no; group size:8–9; phase: II	Mode: treadmill walking, cycle ergometer; supervised: y; intensity: moderate intensity based on individual exercise prescription; session duration: 60 min; RT: y (body-weight, free weights, Therabands, tubing and stability balls)	other components: pt education, psychosocial, risk factor management, nutrition counselling; mode of delivery: f2f: y, tech: no	Gender-tailoring: y (6 principles of women’s health); theoretical basis: y (social-ecological model); proportion of women in sessions: 100%
Reed et al. [73, 74] 2019, Canada; quality: 3/5	Dose: 20 sessions (2×/wk for 10 wks); delivery: cardiologist, physiotherapist (multidisciplinary team: y); open access materials: no; group size: NR; phase: II	Mode: dance; intensity: 4 × 4 min of high-intensity intervals at 85–95% peak HR interspersed with 3 min of low-intensity intervals at 60–70% peak HR; session duration: 45 min; RT: NR	other components: NR but comprehensive; mode of delivery: f2f: y; tech: no;	Gender-tailoring: y (exercise mode); theoretical basis: no; proportion of women in sessions: 100%
Sadeghi et al. [37, 75–77] 2012, Iran; quality: 5/5	Dose: 24 sessions (3×/wk for 8 wks); delivery: physician, nurse, exercise physiologist (multidisciplinary team: y); open access materials: Cds to exercise at home; group size: NR; phase: II	Mode: treadmill walking, cycle ergometer, stair climbing, rowing, step, jogging; session duration: 90 min; RT: y	other components: pt education, psychosocial and nutrition counselling; mode of delivery: f2f: y; tech: y (CDs)	Gender-tailoring: no (but women had another education session regarding CVD risks in women); theoretical basis: no; proportion of women in sessions: 100%
Sengupta et al. [78] 2020, (HerBeat) USA; quality: 4/5	Dose: n/a; delivery: health coach (multidisciplinary team: no); open access materials: no; group size: NR; phase II	Mode: walking; unsupervised; intensity: NR; session duration: n/a; RT: NR	other components: NR; mode of delivery: two f2f and rest are remote by weekly phone calls; tech: y (smartphone-based app)	Gender-tailoring: y (smart phone app targeted to women); theoretical basis: no; proportion of women in sessions: 100%
Shabani et al. [38] 2010, Iran; quality: 4/5	Dose: 36 sessions (3×/wk for 12 wks); delivery: physiotherapist, physician (multidisciplinary team: y); open access materials: no; group size: NR; phase: II	Mode: walking; supervised: y; intensity: started with 40–50% of maximal HR reserve with gradually progressed to 60–80% HR reserve; session duration: 60 min; RT: y (recommended 3 days/wk and consisted of 8–10 exercises covering major muscle group with weight set at 30–40% of 1RM for upper body and 50–60% for lower body)	Other components: NR; mode of delivery: f2f: y; tech: no	Gender-tailoring: no; theoretical basis: no; proportion of women in sessions: 100%
Silber et al. [79] 2015, USA; quality: 3/5	Dose: 36 sessions (1–3 supervised sessions/wk); delivery: dietitian, nurse, or case manager. (multidisciplinary team: y); open access materials: written materials, videos; group size: NR, some 1-1 dietary consultation; phase II	Mode: treadmill walking/jogging, cycle ergometry, and elliptical trainer; supervised: y intensity: aerobic exercise 60–70% of HR reserve, then HIIT was introduced; session duration: 45–60 min; RT: y (10–20 min with 8 to 15 repetitions at intensity of 12–14 RPE, 1–2 sets per muscle group)	other components: pt education, nutrition counseling, weight control, stress management; mode of delivery: f2f: y; tech: y (videos)	Gender-tailoring: no; theoretical basis: no; proportion of women in sessions: ≥ 50%
Szot et al. [80] 2016, Poland; quality: 4/5	Dose:36 sessions (3×/wk for 12 wks); delivery: physician, physiotherapist, nutritionist (multidisciplinary team: y); open access materials: no; group size: 6; phase: NR	Mode: bicycle ergometer; supervised: y; intensity: individual exercise prescription based on treadmill stress test then gradually increasing difficulty and workload; session duration: 90 min; RT:NR	other components: NR; mode of delivery: f2f: y; tech: no	Gender-tailoring: no; theoretical basis: no; proportion of women in sessions: ≥ 50%
Turk-Adawi [25] 2020, International; quality: 5/5	n/a	n/a	n/a	n/a
Tsai et al. [81] 2019, Taiwan; China; quality: 5/5	Dose: 10 sessions; delivery: registered nurse, physician, research assistant (multidisciplinary team: y); open access materials: no (manual ‘Methods for Preventing Cardiovascular Diseases: Living a Healthy Lifestyle’); group size: NR; phase II	Mode: NR; supervised: hybrid (initial f2f introduction of motivational intervention within 3 wks of hospital discharge, then consultation and follow-ups by phone call); intensity: NR; session duration: 90–150 min; RT:NR	other components: pt education through motivational discussion, planning individually tailored lifestyle adjustment and set self-management goals; mode of delivery: f2f and remote both; tech: y (phone calls)	Gender-tailoring: no; theoretical basis: y (motivational); proportion of women in sessions: ≥ 50%
Tyni-Lenne et al. [82] 2002, Sweden; quality: 3/5	Dose: 24 sessions (3×/wk for 8 wks); delivery: cardiologist, physiotherapist (multidisciplinary team: y); open access materials: no; group size: NR; phase: II	Mode: cycle ergometer supervised: y; intensity: 50% of the peak work rate achieved on exercise test; session duration: 60 min; RT: no	other components: NR; mode of delivery: f2f: y; tech: no	Gender-tailoring: no; theoretical basis: no; proportion of women in sessions: 100%
Wojcieszczyk et al. [83, 84] 2012, Poland; quality: 1/5	Dose: 29 sessions (3×/wk for 4 wks, then 2×/wk for 8 wks and 1×/wk for 1 wk); delivery: registered nurse, physiotherapist, physician (multidisciplinary team: y); open access materials: no; group size: NR; phase: II	Mode: Tai Chi, cycle ergometer; supervised: y; intensity: NR; session duration: NR; RT:NR	other components: NR; mode of delivery: f2f: y; tech: no	Gender-tailoring: y (exercise mode); theoretical basis: no; proportion of women in sessions: ≥ 50%

*1-RM* single repetition maximal lift, *Cds* compact discs, *CHD* coronary heart disease, *CR* cardiac rehabilitation, *f2f* face to face, *f2g* face to group, *freq* frequency, *GXT* graded exercise test, *HF* heart failure, *HR* heart rate, *HIIT* high-intensity interval training, *MI* motivational interviewing; n/a, not applicable, *NR* not reported, *pt* patient, *RT* resistance training, *RPE* rated perceived exertion, *SCAD* Spontaneous coronary artery dissection, *SCAD-CR* Spontaneous coronary artery dissection cardiac rehabilitation; tech, technology, *THR* target heart rate, *UK* United Kingdom, *USA* United States of America, *wk* week, *y* yes

^‡^Number yes ratings out of 5 shown

Extrapolating from the International Council of Cardiovascular Prevention and Rehabilitation’s (ICCPR) global CR audit, it is estimated there are 686 programs in 45 countries globally offering some form of women-focused programming [25]; this is 41% of countries that have any CR. However, the nature of what is being offered in the “real-world” is not known. Indeed, there has only been one review in this area, which is published only as an abstract. While this work is an advance, they focused only on randomized trials which may not represent what is available in the real-world. They identified 10 trials, with very little detail provided regarding how they are women-focused.

This also leaves questions regarding whether the nature of women-focused CR as delivered can improve utilization [13] (i.e., are women more likely to enrol if these types of models are available? Are they more likely to adhere and complete the programs?), and whether it does better meet women’s needs (e.g., satisfaction). Therefore, the objectives of this systematic review were to investigate the: (1) nature and (2) availability of women-focused CR (e.g., how delivered, tailoring), as well as (3) effects on (a) utilization and (b) satisfaction.

## Methods

The protocol for this systematic review was registered prospectively on PROSPERO (CRD42020189760). Methods were based on the Cochrane Handbook for Systematic Reviews of Interventions [26]. The review was conducted in accordance with the Preferred Reporting Items for Systematic Reviews and Meta-Analyses (PRISMA) 2020 guidelines [27].

### Inclusion/exclusion criteria

Primary studies of any design, such as randomized trials as well as observational and qualitative studies, were included. In terms of publication type, conference abstracts were included where identified, but the authors were contacted where possible to determine if a full publication was available, and if not, to get further needed details. Theses/dissertations were included. Reviews and editorials were searched to identify primary studies only. Case studies were also excluded.

The CR program had to offer at least initial assessment, structured exercise (supervised or unsupervised), and at least one other strategy to control risk factors [7]. To be considered “women-focused”, CR sessions (e.g., education or exercise components of an overall CR program or peer support) or programs had to include ≥ 50% females. Individually-delivered programs (e.g., home-based or eCR; are generally inherently 100% women) were included if they were tailored to women’s needs or preferences in some way (note this criteria was not specified a priori, but at the time of resolving citation rating discrepancies). We distinguished by phase of CR (i.e., I or inpatient, II or outpatient, and III/IV or maintenance).

Studies that included female adults (ages 18 years or over) with any cardiac conditions were included. The study could have any outcome, given that it was the first review in this area. This paper reports on the outcomes of access, utilization, satisfaction. Clinical, psychosocial and cost outcomes are reported elsewhere [28].

### Search strategy

Eight electronic databases were searched from their inception to May 2020, namely: APA PsycInfo (Ovid), Medline (Ovid), Pubmed (non-Medline), Embase (Ovid), Web of Science Core Collection, Scopus, CINAHL (Cumulative Index to Nursing & Allied Health Literature) (EbscoHost) and Emcare (Ovid). The search strategies were developed in collaboration with an Information Specialist utilizing the PICO framework, subject headings as appropriate for each database, and free-text terms relevant to the topical concepts. No language limits were applied. A sample search strategy for Medline is shown in online (see Additional file 1).

### Study selection

Duplicate citations from the search of the databases were deleted in Mendeley, with the unique citations then imported into Covidence. After training and calibration, two researchers (TM and GMG) independently considered the abstracts of potentially-eligible articles for inclusion. The full-texts of potentially-eligible citations were then considered to ascertain whether they met eligibility criteria. Where unclear, authors were contacted to ascertain whether the CR programs had more than 50% women. For both stages, any disagreements were resolved by the senior researcher (SLG). Once the studies were identified, any related protocol manuscripts, theses/dissertations or publications on the baseline cohort for example were secured to inform data extraction and quality assessment.

### Data extraction and management

Information regarding the study design, sample, nature of the CR program, and outcomes reported were extracted from the included studies. For study design, whether there were any comparison groups was extracted, and if yes, whether they were usual care (UC; i.e., with no CR) or active comparisons (AC; e.g., traditional or home-based CR, components of secondary prevention) groups. In addition, the quality of included studies was assessed using the Mixed-Methods Assessment Tool [29], which is applicable to multiple designs. For each of 5 designs, there are 5 items, which are rated as being present (yes), not present, or indeterminable.

Following training by SLG and GMG, TM independently extracted data for each included study, and rated their quality. A second author (GMG) then independently reviewed the extraction and ratings. Any disagreements were resolved by discussion or, where agreement could not be reached, by consultation with the senior author (SLG; except in relation to quality assessment of studies in which she was involved, to mitigate potential bias).

### Data synthesis

All study results were synthesized tabularly, ordered alphabetically by first author, with key study characteristics summarized. First, the summary of the nature of women-focused CR was qualitative (e.g., gender-tailoring) and quantitative (e.g., sum of studies where CR was all women, whether the women’s aspect comprised the full program or only sessions; as well as whether CR was supervised, group-based, as well as frequency of exercise modes, components, team members). Next, availability information was summarized qualitatively.

For the third objective examining utilization and satisfaction, in accordance with the Synthesis Without Meta-analysis reporting guideline [30], results were grouped by outcome, and then by comparison type (UC or AC, if applicable), with studies of higher-quality design summarized first (i.e., prioritized randomized trials, followed by controlled studies, and then others). Outcome scores at each available assessment point were summarized, as well as tests of effects by group and/or time; Vote counting of significant effects (p-values) by direction was undertaken.

## Results

### Study search and selection

**Fig. 1:**
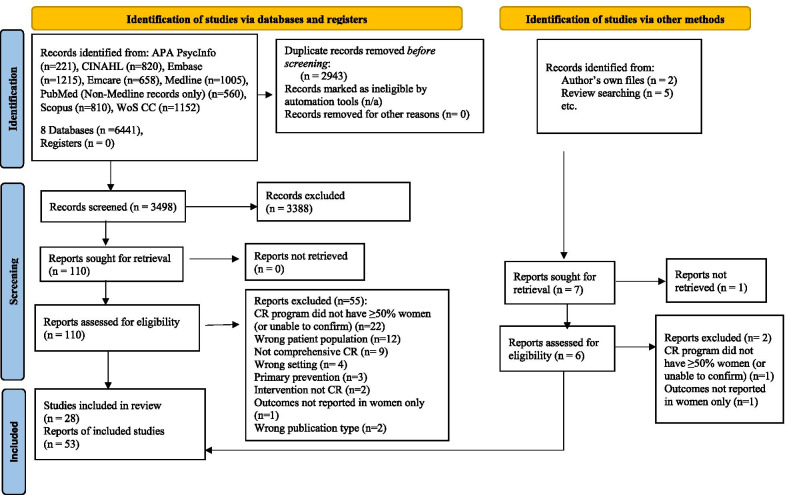
Study selection flow diagram. *APA* American Psychological Association, *CR* cardiac rehabilitation, *CINAHL* Cumulative Index to Nursing & Allied Health Literature, *CVD* cardiovascular diseases, *n/a* not applicable, *WoS CC* Web of Science Core Collection. *From:* Page et al. [27]. https://doi.org/10.1136/bmj.n71. For more information, visit: http://www.prisma-statement.org/

Overall, 3498 unique citations were identified. Upon screening titles and abstracts, four reviews identified were hand-searched [31–34]. No title or abstract was identified in a non-English language which would be considered for full-text review. Four studies from Iran were identified [35–38], and authors were contacted in some instances to confirm female composition and ascertain any tailoring; ultimately these studies were included as they offer only women-only CR there. There was discussion about whether the study by Madison et al. [39], met inclusion criteria of offering structured exercise, but ultimately this study was included. One study undertaken by our group that meets inclusion criteria was recently completed, and was added [40, 41]. Ultimately, 28 studies (53 publications) that met the eligibility criteria were included (Fig. 1) [21, 23, 25, 35–84].

### Study characteristics

As shown in Table 1, the first study was published in 2002. Studies were from the following > 10 countries: 9 (32.1%) from Canada, 5 (17.9%) from the United States of America, 4 (14.3%) from Iran, 2 (7.1%) from United Kingdom, 2 (7.1%) from Poland, 2 (7.1%) from Sweden, 1 (3.6%) was international [25], and 1 (3.6%) each stemmed from Australia, Taiwan/China), as well as Saudi Arabia. The Southeast Asian and African regions are not represented (likely reflective of the fact that both have very little CR) [85]. Only Iran is a non-high-income country [86]. Six (21.4%) studies were multi-centre; not considering the global audit of women-focused CR [25], ultimately 44 centers were studied.

With regard to design, 11 (39.3%) of the included studies were randomized trials, some with more than 2 arms. Five (45.4%) had UC control arms; Of those 9 with AC, these included: traditional supervised CR, hybrid models (i.e., supervised sessions transitioning to remote), home-based CR, structured exercise only, education only, Tai Chi, relaxation therapy, and cognitive-behavioral psychotherapy [83, 84]. The remaining 17 studies were prospective cohort (n = 14, 82.4%; of which n = 8 had control groups), mixed-methods (n = 2, 11.8%) or descriptive (n = 1, 5.8%) in design. Duration of follow-up ranged from 1 to 60 months (5 years [42]), with a median of 3 months.

Quality of each study is also shown in Table 1. The median number of yes’ (indicating good quality) per study was 4/5.

### Participants

Sample sizes across studies ranged from 9 to 727 participants, with a median of 60. In all but 2 (92.9%) studies, the samples were comprised of only female participants; in the other two studies, women were compared with a sample of male participants [40, 41, 76]. Overall, there were 3697 women studied.

Mean age of included women participants was 59.3 ± 7.1 years (standard deviation). Ethnocultural background was reported in 7 (25.0%) studies, which mostly comprised white caucasians. Most participants (41.1%) had coronary artery disease, however other diagnosis more common in women were also represented such as: spontaneous coronary artery dissection (SCAD; 2 studies with 79 women [54, 79]), ischemia with no obstructive coronary artery disease (INOCA; 3 studies with 143 women [44, 80, 82]), and heart failure only (2 studies with 83 women [45, 61]).

### Nature of women-focused CR

Twenty-three (82.1%) studies tested phase II (outpatient) CR, 3 (10.7%) were phase III [35, 39, 44], and one study covered phases I, II and III [60]. In 22 (78.6%) studies, CR was delivered in a clinical setting such as a hospital only, and in 5 (16.1%) it was primarily remote (e.g., home-based, technology) (Table 1; 1 study assessed availability so not listed here) [25]. In 9 (32.1%) studies, some form of technology was used (e.g., telephone, remote monitoring, smartphone apps). CR was offered individually rather than in groups in 2 (7.1%) studies (i.e., home-based, in-person [61]; in-person for initial orientation then complete the program at home [55]).

There was a range of 4–56 sessions/program overall (median = 24). Thirteen (46.4%) studies offered only women-focused sessions (with an average of 14.2 such sessions/program, where reported) [23, 39–41, 43, 46, 54, 55, 59, 61, 64, 68, 73, 78], with the remainder of studies testing full women-focused programs (Table 1).

All CR programs incorporated aerobic exercise of some mode (i.e., treadmill, cycle ergometer, walking or stair climbing); 10 (35.7%) had resistance exercise. In 5 (17.9%) studies, there were alternative forms of exercise (e.g., Tai Chi, dance, aerobics, yoga, Qigong, water aerobics; Table 1). Seventeen (60.7%) studies had a psychosocial component (e.g., meditation, progressive muscle relaxation, cognitive-behavioural techniques). Types of healthcare providers delivering services are shown in Table 1, with 23 (82.1%) studies having more than 1 profession (i.e., multidisciplinary team).

With regard to how interventions were designed to meet women’s needs (Table 1), in 17 (60.7%) studies the CR included no men (in the 4 studies from Iran, as outlined above, women only participate in women-only programs for cultural reasons [35–38]) and 11 (39.3%) had some form of gender-tailoring of content other than form of exercise as outlined above (with many studies having both, and others mostly women with or without tailoring). With regard to the latter content, in 9 (32.1%) studies, education was tailored to consider women-specific information needs, such as regarding risk factors, forms of CVD and comorbidities more common in women.

### Availability, utilization and satisfaction with women-focused CR

Availability of women-focused CR sessions or programs around the world was summarized in the introduction [25]. In that 2016 audit, women-focused CR was estimated to be available in 45 countries. Through this review we identified Sweden, Taiwan and Saudi Arabia also offer it. The audit revealed women-focused CR programs were most commonly-available in the Eastern Mediterranean region and Europe, with North America relatively more represented in this sample. On average, 1/3 of programs in a country with women-focused CR offered it.

With regard to CR accessibility, a qualitative study reported that the process of learning about the women-only program and obtaining physician referral were among the barriers in accessing women-only CR [70]. Two studies from Toronto identified that women-focused CR may be less accessible because it inherently cannot be offered as frequently as mixed-sex CR. For instance, in the CR4HER trial, many women did not follow random allocation and switched from women-only to mixed-sex program models; semi-structured interviews revealed this was due to time conflicts with the only one available time per week the women-only program sessions were offered [21]. In the other study at the same centre, participants could elect women-only, mixed sex or home-based models. Only 22.0% elected women-only, and they were less often working, so likely had more time flexibility [40].

With regard to CR utilization, first, enrolment (i.e., attendance at initial visit) was considered. In Beckie et al.’s trial, 89.2% initiated the traditional mixed-sex CR and 97.2% initiated the women-focused program [47]. In Azad et al.’s trial, of 51 heart failure participants randomized to the women-focused CR group, 8 dropped out (84.3% enrolment; no comparative data available). This was not reported by group in most studies, and in no studies was it inferentially tested by group, so receipt of allocated intervention was extracted from all trials as a proxy for descriptive purposes at the least (Table 2). For example, in the CR4HER trial, 63.6% of women allocated to women-only CR, 67.8% allocated to mixed-sex and 43.6% allocated to home-based CR attended the initial visit at CR in their allocated model [64]. Overall, “enrolment’ in women-focused CR arms was on average 93.7%, and in AC arms was 87.2%.

**Table 2:** Randomized women-focused CR trial design, and summary of utilization (N = 11), plus availability study

Study author, year, country	Nature of comparison arm(s); # centres	Participants/sample: size (% female), mean age; ethnocultural background; CHD type [& % HF]; males for comparison (y/n)	Results
Andersson et al. [42], 2010; Sweden	AC: physiotherapy (8 sessions = 2×/wk for 4 wks, bicycling or aerobic exercise; information on healthy food and adverse effects of nicotine provided); 1 centre	N = 149 (100% female); mean age: 53.4 ± 6.2 yrs; ethnocultural background: NR; CHD type: MI (65.2%) (& 0% HF); Males for comparison: no	NR
Arthur et al. [43] 2007; Canada	AC: AT (48 sessions = 2×/wk for 24 wks, 40 min; moderate intensity; using stationary cycles, treadmills, arm ergometers, stair climbers; received other components of comprehensive CR); 1 centre;	N = 92 (100% female); mean age: NR; ethnocultural background: NR; CHD type: MI (& 0% HF); Males for comparison: no	*Women-focused CR*: 46 randomized, 42 (91.3%) enrolled, 37 (80.4%) completed; AC: 46 randomized, 40 (86.9%) enrolled, 35 (76.1%) completed
Asbury et al. [44] 2008; UK	UC control (with symptom monitoring only); 1 centre;	N = 64 (100% female); mean age: 57.3 ± 8.6 yrs; ethnocultural background: NR; CHD type: cardiac syndrome X (& 0% HF); Males for comparison: no	*Women-focused CR*: 32 randomized, 30 (93.8%) enrolled, 28 (87.5%) completed
Beckie et al. [46–53] 2010; USA	AC: Traditional CR (36 sessions = 3×/wk for 12 wks; aerobic training by treadmill walking, cycling or rowing; eight education classes of 1 h duration on CHD risk factor modification before each exercise session); 1 centre	N = 252 (100% female); mean age: 61.6 ± 10.0 yrs; ethnocultural background: caucasian 82.0%; CHD type: MI (4.4%), chronic SA (12%), (& 0% HF); Males for comparison: no	*Women-focused CR*: 141 randomized, 137 (97.2%) enrolled, 133 (94.3%) completed; *AC*: 111 randomized, 99 (89.2%) enrolled, 99 (89.2%) completed
			*Mean number of 36 exercise sessions attended*: Women-focused CR 32 ± 9; AC 28 ± 12; Significant difference between the two groups (p < 0.001)
			*Mean percent attendance at education sessions*: Women-focused CR 87 ± 24; AC 56 ± 30; Significant difference between the two groups (p < 0.001)
Clark et al. (Women Take Pride trial) [55–58] 2003; USA	AC: women tailored group format (7 sessions = 1×/wk for 6 wks, then at 6 months another session, all f2f, 6–8 women/group); UC (routine care with physician); multi-centre (12)	N = 575 (100% female); mean age: 72.8 ± 7.9 yrs; ethnocultural background: caucasian 82.8%; CHD type: MI (41.7%), SA (37.6%), (& 23% HF); Males for comparison: no	*Women-focused CR*: 201 randomized, 197 (98.0%) enrolled, 164 (81.6%) completed; *AC*: 190 randomized, 185 (97.3%) enrolled, 166 (87.4%) completed
Feizi et al. [35] 2012; Iran	AC1: PMR (2 f2f sessions, 16-muscle groups, then practice PMR 15 min daily at home) AC2: phase III CR (with aerobic exercise including walking with gradually increasing intensity and duration of maximum 40 min; stretching, educational pamphlet and Cds also provided to practice) vs UC [no CR or PMR]); 1 centre;	N = 40 (100% female); mean age: 50.9 ± 6.9 yrs; ethnocultural background: NR; CHD type: cardiac syndrome X (& 0% HF); Males for comparison: no	*Women-focused CR*: 11 randomized, 11 (100.0%) enrolled, 11 (100.0%) completed; *AC1*: 11 randomized, 11 (100.0%) enrolled, 11 (100.0%) completed; *AC2*: 11 randomized, 11 (100.0%) enrolled, 11 (100.0%) completed
Gary et al. [61–63] 2003; USA	AC: education-only control (received 1×/wk home visits for 12 wks); 1 centre;	N = 32 (100% female); mean age: 68.0 ± 11.0 yrs; ethnocultural background: caucasian 59.3%; CHD type: 100% HF; Males for comparison: no	*Women-focused CR*: 16 randomized, 16 (100.0%) enrolled, 15 (93.8%) completed; *AC*: 16 randomized, 16 (100.0%) enrolled, 13 (81.3%) completed
Grace et al. (CR4HER trial) [21, 64–67] 2014; Canada	AC1: supervised mixed-sex CR (48 sessions = 2×/wk for 24 wks, 60 min; aerobic exercise via stationary bicycle/treadmill/walking and education classes); AC2: home-based CR (27 sessions = 3 supervised and 1×/wk for 24 wks phone calls along with education materials); 3 centres	N = 169 (100% female); mean age: 63.64 ± 10.42 yrs; ethnocultural background: caucasian 62.5%, CHD type: AMI (35.8%), (& 0% HF); Males for comparison: no	*Women-focused CR*: 55 randomized, 35 (63.6%) enrolled, 59.94% (SD: NR) session adherence, 21 (38.2%) completed; *AC1*: 59 randomized, 40 (67.8%) enrolled, 65.51% (SD: NR) session adherence, 21 (35.6%) completed; *AC2*: 55 randomized, 24 (43.6%) enrolled, 75.32% (SD: NR) session adherence, 20 (36.4%) completed
			There was a significant difference in CR adherence by program model (p < 0.001). Home-based CR participants adhered to a significantly higher percentage of sessions than participants in women-focused CR (post-hoc LSD test, p = 0.03)
Turk-Adawi [25] 2020; International	Descriptive, global CR audit and survey	203 countries in world; 111 (54.7%) offer CR; data collected in 93 (83.8%); n/a	Thirty-eight (40.9% of those offering CR) countries with CR offered women-only CR globally (18.7% of all countries globally)
			Overall, in countries that delivered it, on average 32.1% programs offered women-only CR. In Iran, Pakistan and Greece, it was delivered in > 50% of programs
			Provision of women-focused CR was greater in EMR region. Countries in the Western Pacific region had the lowest proportion of programs (1.2%)
			Programs that offered women-focused CR were more often: located in an academic or tertiary facility, served more patients/year, offered more components, treated more patients/session, offered alternative forms of exercise, had more staff (including cardiologists, dietitians, and administrative assistants, but not mental health care professionals), and perceived space and human resources to be less of a barrier to delivery than programs not offering women-focused CR (all p < 0.05), suggesting it is only feasible for larger, well-resourced programs to offer it
Tsai et al. [81] 2019; Taiwan, China	UC: received regular health education; 2 centres;	N = 35 (100% female); mean age: 56.1 ± 5.6 years; ethnocultural background: NR, CHD type: coronary artery stenosis; 0% HF. Males for comparison: no	*Women-focused CR*: 17 randomized, 17 (100.0%) enrolled, 16 (94.1%) completed
Tyni-Lenne et al. [82] 2002; Sweden	AC: relaxation therapy [16 sessions = 2×/wk for 8 wks, 60 min; consisted of modified Jacobson’s approach and autogenous training], UC: normal daily activities; 1 centre;	N = 24 (100% female); mean age: 55.0 ± 8.0 years; ethnocultural background: NR, CHD type: cardiac syndrome X. (& 0% HF). Males for comparison: no	*Women-focused CR*: 7 randomized, 7 (100.0%) enrolled, 6 (85.7%) completed; *AC*: 7 randomized, 7 (100.0%) enrolled, 6 (85.7%) completed
Wojcieszczyk et al. [83, 84] 2012; Poland	AC1: Traditional CR (29 sessions = 3×/wk for 4wks, then 2×/wk for 8 wks, then 1×/wk for 1 wk; cycle ergometer), AC2: Traditional CR and cognitive behavior psychotherapy; 1 centre	N = 68 (100% female); mean age: 62.07 ± 6.00 years; ethnocultural background: NR, CHD type: MI (& 0% HF). Males for comparison: no	*NR*

When program utilization data were not available, information from each assessment point was extracted as a proxy

*AC* active comparison, *AC1* active comparison control group 1, *AC2* active comparison control group 2, *AT* aerobic training, *CDs* compact discs, *CHD* coronary heart diseases, *CR* cardiac rehabilitation, *HF* heart failure, *LSD* least significant difference, *MI* myocardial infarction, *NR* not reported, *UC* usual care, *n/a* not applicable, *PMR* progressive muscle relaxation, *SA* stable angina, *SD* standard deviation, *wks* weeks

With regard to program adherence (or percentage of prescribed sessions attended), 5 studies reported on this, including 2 trials which will be summarized first. In Beckie et al.’s trial in the United States, adherence to the 36 women-only sessions with motivational interviewing was significantly greater than traditional CR [51]. In the CR4HER trial, adherence to all models was modest (54.5%), and did not differ by model on a per-protocol basis; as-treated, it appeared that women were more likely to adhere to home-based than either women-only or traditional supervised CR, however this should be interpreted with caution as women can more easily participate in CR phone calls than travel for site visits, and there were fewer calls in the home-based model than site visits in the supervised models [64].

In non-randomized studies, one Canadian study showed women enrolled in mixed-sex CR adhered to a significantly *greater* proportion (58.8 ± 28.9% of sessions attended/25) of prescribed sessions compared to women-only (54.3 ± 26.3% of sessions attended/25) [40]. Azad et al. reported high program adherence in women with heart failure at 87% of prescribed sessions [45]. In another Canadian study, mean adherence to a women-only model was 75.7% of sessions, with significantly greater use of CR services (e.g., nutrition, exercise, nursing) among women-only participants compared to matched women in their traditional mixed-sex model [68].

In trials where it was reported, CR completion in women-focused and AC arms were on average 83.9% and 76.9%, respectively (Table 2). Two non-randomized studies reported on CR completion (i.e., attendance at post-program assessment). Again in the Canadian, program completion was significantly greater in both supervised models (i.e., equivalent in women-only and mixed-sex models) when compared to home-based; the authors surmised this was due to the fact that women electing home-based services would not be readily-available to come on site for a post-program assessment [40]. Another study in Saudi Arabia reported a women-only CR completion rate of 54.3% [60].

With regard to satisfaction, there was 1 study assessing this outcome. In the CR4HER trial, participants were significantly more comfortable in their workout attire and perceived the environment as less competitive in the women-only program compared to traditional CR [21]. Yet, ratings of satisfaction were high across all models. Model preference did not differ between mixed-sex and women-only (41.9%, although this was much higher than for home-based at 16.2%); Ultimately, women preferred the model they attended however.

## Discussion

To our knowledge, this is the first full review investigating the nature of women-focused CR, seeking to establish global availability, common features, access/utilization by women, and satisfaction. Women-focused CR is not widely available; as established, any CR is insufficiently available [85], and in the countries where CR is available, only about 40% have any women-focused CR, with most programs in the countries not offering it. This renders women’s-focused CR highly inaccessible, particularly given less than 1/3 of the programs used any form of technology so could not reach women beyond their locality (if women had the hardware and proclivity to do so, which requires further study).

It could be this limited availability that explains why so few women are aware of women-focused CR (they are hardly aware any CR exists) [19, 87], and hence be aware they can access it [70]. Accessibility is also limited in that it is offered less frequently than traditional mixed-sex CR [21]. This can be particulary problematic for women who are working or have caregiving responsibilities [40], hence offering women-focused programming virtually or asynchronously should be explored.

**Fig. 2:**
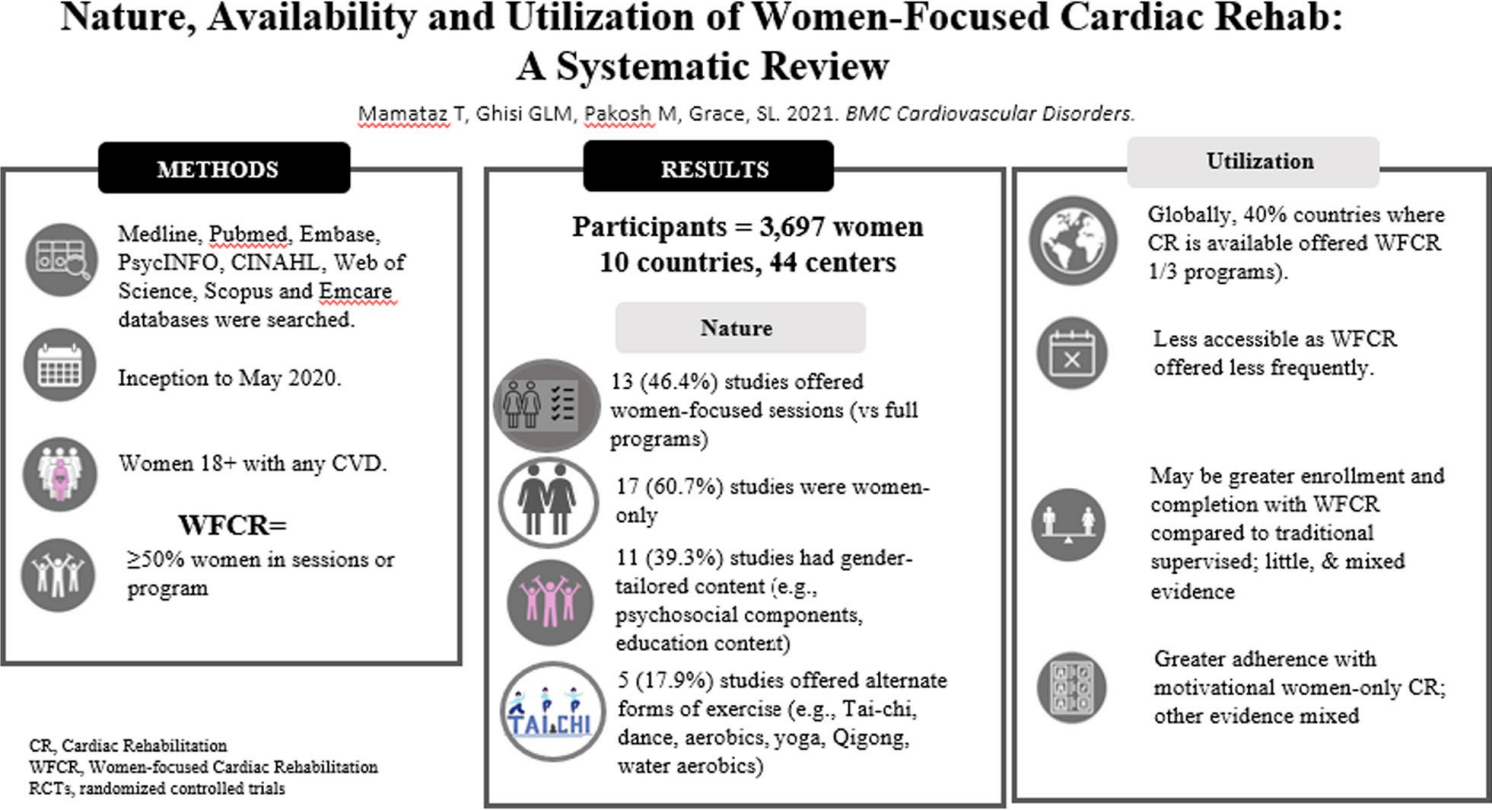
Summary of results

As summarized in Fig. 2, in about half of studies, the women-focused programming was delivered across the full program, and for the remainder it was a part. In 60% of studies, the CR intervention was for women only, and for the remainder men were involved as well. Only a third had content tailored to women, most commonly education or psychosocial programming. Indeed, about 60% had any psychosocial component, despite desire for this in women [20]. In less than one-fifth of studies were non-traditional forms of exercise offered, again despite this preference by women [20]. This was surprising, and suggests there may be challenges for programs in safely, affordably and/or equitably offering such programming; future research should investigate program-level barriers to offering modes of exercise preferred by women. Finally, given women often desire social interaction [20], it was appropriate most programming was delivered in a group; it may be desirable to do this virtually more commonly in future to augment accessibility for women [78]. It is hoped with this information the CR community can come to consensus on what is considered women-focused CR, with our suggestion that it refers to programs: (a) with at least some CR components with ≥ 50% women, and (b) comprising some form(s) of tailoring to meet women’s needs or preferences (e.g., fulsome psychosocial screening and programming, education content, and/or forms of exercise). Moreover, (c) setting/mode of delivery (i.e., to address women’s common transportation barriers and time constraints related to caregiving responsibilities, respect their preference for more privacy) as well as (d) clinician sex, disciplines represented on the team (e.g., staff with specialization in women and CVD, and in mental health), who deliver patient-centered care for women [88–90], should also be considered.

Women-focused CR delivery materials were provided in few studies unfortunately, hampering replication. While practical direction on how to develop women-focused CR is needed, some preliminary insights can be gleaned in the literature. Notably, Price et al. applied 6 principles for women’s health to inform their women-only and tailored CR program development [23]. The principles included: empowering women (e.g., encouragement to strengthen personal resources, personal goal setting, empathic environment), that health services are defined broadly and are accessible (e.g., evening classes, cultural sensitivity), that care is collaborative and patient-centered [88–90], and that innovative and creative approaches are used to meet women’s needs. A narrative review is available on ways in which community and home-based programs could better meet women’s needs, such as offering appealing forms of exercise [91], offering flexible timing and setting, as well as promoting social interactions [24]. With the COVID-19 pandemic, our program delivers weekly women-focused CR sessions, based on our validated patient education program [92–94], which are freely available online (https://www.healtheuniversity.ca/EN/CardiacCollege/Pages/Women-Learn-Online.aspx). A recently-published paper presents development and evaluation of a theoretically-based mobile phone-based women-focused CR program using machine learning [78]; preliminary engagement and walking results are promising.

With regard to adherence to these programs once women access them, the findings are mixed (Fig. 2). Overall, results seem to suggest that women are more likely to adhere where programs have some form of tailoring [51], and it may not be the “women-only” aspect that makes a difference [40, 64, 68]. Given women do not like to experience pain or fatigue with exercise, important questions regarding adherence to exercise related to intensity of prescription and exercise mode also require study. There is grossly insufficient data on differences in program completion. Clearly, more research in this area is needed. Interestingly however, a recent study in Sweden found higher mixed-sex CR enrolment among women than men, which they attributed to the fact that it is one of the most gender-equal countries in the world [95], and hence “traditional” CR may also by design better meet women’s needs and preferences. This supports that perhaps CR need not be women-only, but women-tailored.

Unfortunately, only 1 trial with satisfaction data was identified, which assessed a women-only and not gender-tailored program; results were equivocal [21]. Perhaps with this first information regarding what is being offered in women-focused CR, as a CR community we can better standardize women-focused CR feature categorization, and start testing what features are related to greater program satisfaction. Development of a consensus statement on best practices could be useful for the field, until more needed research accrues.

Indeed, this review has raised many questions. Several directions for future research have been identified above. Overall, while it is encouraging that 11 trials have now been done in this area, there is little controlled data on utilization and satisfaction. In addition, while women-focused CR was most often delivered in a clinical setting (which is likely also a function of our inclusion criteria), given home-based CR is inherently “women-only”, more research on ways it can be tailored to better meet women’s needs is needed. Indeed, home-based CR can mitigate some of women’s barriers to utilization, and additionally some women prefer it [22]. Although, home-based CR has been historically less available than traditional CR [96], often likely because it is not as commonly reimbursed [97], with the COVID-19 pandemic there has been a massive shift to online delivery [98], with associated advocacy for coverage, rendering this a more possible avenue in future.

If the evidence does warrant it, what kind of women-focused CR should be scaled up? Based on current knowledge and practice, offering group women-only sessions (not full programs), virtually, in the evenings, tailored to women’s psychosocial and educational needs is likely advisable. Based on the evidence, recommendations regarding dose cannot be made, but leveraging peer support could also meet women’s preferences, while reducing workload on CR staff to deliver such specialized programming. Encouraging women to engage in their preferred exercise modality outside of CR would also be helpful, if programs do not have the capacity to offer it; community resource lists could be developed and shared with women [99]. Exploiting technology could expand reach and contain costs, however patient-centred care is still needed, including close attention to clinical status. Finally, considering context would also be important; culture has an impact on women’s health behaviours and healthcare utilization [100].

While quality of included studies was generally acceptable, caution is necessary when interpreting the results of this review. First, data extraction was not done independently by two researchers. Second, there was no grey literature search, nor were any trial registries searched for potential studies to include. Third, generalizability is limited. Studies were of small sample size, but cardiac conditions more common in women were represented. Given the estimated availability of CR globally [85], studies identified herein stem from approximately 25% of the countries that offer women-focused programming, and 10% of programs [25]. For example, women-focused CR is known to be available in South Africa, Brazil, Chile, Colombia, Paraguay, Uruguay, Afghanistan, Bahrain, Pakistan, Qatar, Belarus, Bosnia, Czech Republic, Turkey, India, Indonesia, mainland China, Malaysia, as well as several European countries [25], yet we do not know what is offered there. Programs in the Eastern Mediterranean in particular were less well-represented in the sample, given the preponderance of women-only programming is delivered there [101]. As women-only CR is culturally-prescribed in the region commonly, more understanding of what is delivered there is urgently needed [100].

In conclusion, half the time women-focused CR involves full programs, including only women, and offering a psychosocial component; as well, in a third of programs content is gender-tailored, and some offer alternative modes of exercise preferred by women. More research is needed to establish the features of women-focused CR that are associated with greater use, satisfaction and outcomes. Given it is not often available, programs may wish to consider offering women-focused sessions virtually, with peer support, addressing women’s unique CVD features and psychosocial needs.

## Supplementary Information

**Additional file 1.** Search Appendix.

## Data Availability

The data used and analysed in this review are publicly-available and shown in the display items.

## Abbreviations

AC: Active comparison
CR: Cardiac rehabilitation
ICCPR: International Council of Cardiovascular Prevention and Rehabilitation
INOCA: Ischemia with non-obstructive coronary arteries
PRISMA: Preferred Reporting Items for Systematic Reviews and Meta-Analyses
SCAD: Spontaneous coronary artery dissection
UC: Usual care
