# Supplementary material for: Nature, availability, and utilization of women-focused cardiac rehabilitation: a systematic review
Source: BMC Cardiovasc Disord. 2021 Sep 23;21:459. doi: 10.1186/s12872-021-02267-0 (PMC8458788; doi:10.1186/s12872-021-02267-0)
Supplement: Supplementary file 1 — Additional file 1. Search Appendix. [file 12872_2021_2267_MOESM1_ESM.docx]

# Additional file 1

Search Strategy Ovid MEDLINE(R)

ALL <1946 to May 21, 2020>

--------------------------------------------------------------------------------

1 exp Heart Diseases/ (1118129)

2 Cardiac Rehabilitation/ (2289)

3 ((cardiac or heart or coronary) adj3 (disease* or disorder* or anomol* or disturbance* or deficien* or deformit* or dysfunction* or patient*)).tw,kw. (433296)

4 (rehab* adj3 (cardiac or cardiovascular or heart or coronary or infarct* or myocardi*)).tw,kw. (8098)

5 or/1-4 (1270637)

6 exp Women/ (36227)

7 Women's Health/ (27405)

8 Women's Health Services/ (3826)

9 ((women* or woman*) adj4 (only or prefer* or suit* or tailor* or satisf* or among or design* or participa* or feasible or usable or utili?e or underutili?e or under-utili?e or availab* or class* or session* or program* or barrier* or attend* or needs or perception*)).tw,kw. (195004)

10 or/6-9 (243445)

11 Cardiac Rehabilitation/ (2289)

12 exp Exercise/ (192756)

13 exp Exercise Therapy/ (50083)

14 exp Exercise Movement Techniques/ (8112)

15 cardiorespiratory fitness/ (1473)

16 Self-Management/ (2055)

17 ((self-manag* or self manag* or self-monitor* or self monitor*) adj4 (heart* or cardiac or program*)).tw,kw. (2942)

18 (exercis* or kinesiotherap* or kinesitherap*).tw,kw. (296864)

19 (physical adj3 (training or conditioning or fitness)).tw,kw. (18704)

20 (training adj4 (resistanc* or weight* or strength* or interval* or intermittent* or cardio*)).tw,kw. (24709)

21 (walk* or run or running or jog or jogging or swim* or bicycl* or treadmill*).tw,kw. (326088)

22 (rehab* adj3 (cardiac or cardio*)).tw,kw. (7506)

23 (cardio* adj3 fitness).tw,kw. (7088)

24 or/11-23 (663136)

25 5 and 10 and 24 (1005)

***************************
